# The New Year

**Published:** 1854-01

**Authors:** 


*The New Year.*—Not only in accordance with honored custom, and not merely to comply with a required formality, but with a full sense of the meaning of the wish, and a hearty hope that it may be realized, we invoke for all our readers, "A Happy New Year."

